# The Tanita SC-240 to Assess Body Composition in Pre-School Children: An Evaluation against the Three Component Model

**DOI:** 10.3390/nu8060371

**Published:** 2016-06-16

**Authors:** Christine Delisle Nyström, Pontus Henriksson, Christina Alexandrou, Marie Löf

**Affiliations:** 1 Department of Biosciences and Nutrition, Karolinska Institutet, 141 83 Huddinge, Sweden; alexandrou81@gmail.com (C.A.); marie.lof@ki.se (M.L.); 2 PROmoting FITness and Health through Physical Activity Research Group (PROFITH), 18071 Granada, Spain; pontus.tm.henriksson@gmail.com; 3 Department of Clinical and Experimental Medicine, Faculty of Health Science, Linkoping University, 581 83 Linköping, Sweden

**Keywords:** Tanita SC-240, bioelectrical impedance, three component model, pre-school

## Abstract

Quick, easy-to-use, and valid body composition measurement options for young children are needed. Therefore, we evaluated the ability of the bioelectrical impedance (BIA) device, Tanita SC-240, to measure fat mass (FM), fat free mass (FFM) and body fatness (BF%) in 40 healthy, Swedish 5.5 years old children against the three component model (3C model). Average BF%, FM, and FFM for BIA were: 19.4% ± 3.9%, 4.1 ± 1.9 kg, and 16.4 ± 2.4 kg and were all significantly different (*p* < 0.001) from corresponding values for the 3C model (25.1% ± 5.5%, 5.3 ± 2.5 kg, and 15.2 ± 2.0 kg). Bland and Altman plots had wide limits of agreement for all body composition variables. Significant correlations ranging from 0.81 to 0.96 (*p* < 0.001) were found for BF%, FM, and FFM between BIA and the 3C model. When dividing the children into tertiles for BF%, 60% of children were classified correctly by means of BIA. In conclusion, the Tanita SC-240 underestimated BF% in comparison to the 3C model and had wide limits of agreement. Further work is needed in order to find accurate and easy-to-use methods for assessing body composition in pre-school children.

## 1. Introduction

Childhood overweight and obesity is a serious issue affecting around 42 million children under the age of five [1]. This is of great concern because children who are overweight or obese have an increased likelihood of developing health problems such as diabetes and cardiovascular disease earlier in life than their normal-weight peers [1]. Overweight and obesity is characterized by a large accumulation of body fat [2] and body mass index (BMI) is the most common way to classify people into the categories of underweight, normal weight, overweight, and obese. Ellis *et al.* [3] found that there was a large range in body fat percentage (BF%) for each BMI category and concluded that BMI is not an adequate measure of obesity in children. A recent study in Swedish four years old also found that BMI does not adequately classify children with a high BF% as measured via air displacement plethysmography (ADP) [4]. Therefore, easy and accurate methods for assessing body composition early in life are needed to identify children with high BF%.

There are a variety of techniques available to measure body composition; however, each method comes with its own unique set of advantages and disadvantages. Multicomponent models, more specifically the three or four component models are suggested to be criterion methods for assessing body composition [5]. The three component model (3C model) divides the body into three components, fat mass (FM), dry fat free mass (FFM) and water; whereas, the four component model (4C model) creates a fourth component by dividing dry FFM into proteins and minerals [5]. Two component models (2C models) such as ADP and isotope dilution divide the body into two components, FM and FFM which are calculated from measured body volume and total body water (TBW) respectively. Dual energy X-ray absorptiometry (DXA) measures bone mineral, lean body mass, and fat which can be used to calculate FM and FFM. Although these methods are generally considered to be quite accurate, they can be expensive, time intensive, and require subjects to come to a measurement at a specific location.

Bioelectrical impedance analysis (BIA), which is also a 2C model can estimate FFM through estimating TBW using the conductive properties of the body’s tissues [5]. It is easy-to-use, inexpensive, and transportable which allows it to be easily used in studies where a large number of participants are measured. There are numerous different types of BIA devices, which all need to be validated in the desired population before use. The foot-to-foot Tanita SC-240 body composition analyzer’s ability to assess body composition has been evaluated previously in 5 to 18 years old children using DXA and no significant difference in BF% was found between the methods [6]. These results are in contrast to previous results using foot-to-foot BIA and DXA in children where significant differences between these two methods have been found [7,8]. Hence more studies are needed to evaluate the foot-to-foot BIA in young children.

The mobile-based intervention intended to stop obesity in preschoolers (MINISTOP) study is a population based randomized controlled trial that aims to determine the effectiveness of a 6-month mobile phone based intervention to improve body composition, dietary habits, physical fitness, physical activity, and sedentary behavior in healthy pre-school aged children [9]. This nested validation study within the MINISTOP trial was conducted to evaluate the ability of the Tanita SC-240 body composition analyzer to measure body composition in healthy, Swedish 5.5 years old against the 3C model.

## 2. Materials and Methods

### 2.1. Participants and Study Design

Participants for the MINISTOP trial were recruited from a population based sample created using the population registry at Statistics of Sweden [9]. At the second follow-up in the MINISTOP trial which began in February 2015, parents and their children were asked to participate in a nested validation of dietary intake [10], physical activity, and body composition methods. All returning parents and children were asked to participate until 40 agreed to do so, with 45 parents being asked in total. Parents brought their child to the Linköping University Hospital for anthropometric and body composition measurements. The parents were instructed not to give food and drink to their child within close proximity to the measurement period. Characteristics of the study sample in regards to age, weight, height, and BMI were comparable with those in the whole MINISTOP trial (*n* = 315).

This study was approved by the Research and Ethics Committee, Stockholm, Sweden (2013/1607-31/5; 2013/2250-32) and informed consent was obtained from all of the parents. The MINISTOP trial is registered as a clinical trial (https://clinicaltrials.gov/ct2/show/NCT02021786).

### 2.2. Measures

#### 2.2.1. Anthropometry

The weight and height of the children were collected using a digital scale and wall stadiometer when the children were wearing light clothing and no shoes. Weight and height were recorded to the nearest 0.1 g or cm, respectively [11]. BMI was calculated as weight (kg)/height (m^2^) and the International Obesity Task Force’s BMI cut-offs were used to classify children as overweight or obese [12]. Weight for age and height for age z-scores were calculated using Swedish reference data [13].

#### 2.2.2. Bioelectrical Impedance

The Tanita SC-240 foot-to-foot body composition analyzer (Tanita Cooperation, Tokyo, Japan) was used to assess bioelectrical impedance. Measurements were collected at 50 Hz using the standard setting after manually imputing the measured height, gender, and age of the subject. The children were bare foot and wore minimal clothing and were instructed to standstill with their feet touching all four metal plates. BF% was then estimated using the in-built Tanita equations. FM (kg) was calculated as: BF% divided by 100 and then multiplied by body weight and FFM (kg) was subsequently calculated as the difference of body weight and FM.

#### 2.2.3. Three Component Model

The 3C model [14] was used as the criterion reference method in this validation. The pediatric option for BodPod (COSMED USA Inc., Concord, CA, USA) was used to assess body volume by means of ADP as we have previously described [4,15]. Isotope dilution was used to assess TBW. All 40 children were provided with an accurately weighed dose of stable isotopes (0.14 g ^2^H_2_O and 0.35 g H_2_^18^O per kg of body weight) as previously described [10]. Urine samples before and after the dose were collected, stored, and analyzed for isotope enrichments using isotope ratio mass spectrometry as published previously [10]. Deuterium (^2^H) dilution space (N_D_) and oxygen-18 (^18^O) dilution space (N_O_) were determined using zero time enrichments attained from the exponential disappearance curves that provided estimates for the elimination rates for ^2^H and ^18^O, respectively. N_D_/N_O_ was 1.039 ± 0.008 for all 40 children. TBW was calculated as the average of N_D_/1.041 and N_O_/1.007 [16]. FM was calculated using the following equation:

FM (kg) = [(2.220 × BV) − (0.764 × TBW)] − (1.465 × BW)

BV equals body volume (L), TBW equals total body water (L), and BW equals body weight (kg). FFM (kg) was calculated as the difference between body weight and FM and BF% was calculated as FM (kg) divided by body weight multiplied by 100.

### 2.3. Statistical Analysis

Values are given as means and standard deviations (SD). Significant differences between mean values were identified using paired sample *t*-tests. The Bland and Altman method [17] was used to compare BF%, FM, and FFM determined using BIA *versus* the 3C model. Using the Bland and Altman method, BF%, FM, or FFM assessed using BIA minus BF%, FM, or FFM acquired via the 3C model (*y*-axis) were plotted against the average of BF%, FM, or FFM assessed via BIA and the 3C model (*x*-axis). The mean difference as well as the limits of agreement (±2SD) were then calculated. To test for a trend between the two methods, a linear regression model was fitted between the *x* and *y* axis. Pearson correlation analyses were then conducted to assess the relationship between the variables.

Classification capacity of the BIA was evaluated against the 3C model by ranking the subject’s BF% in a sequence. Thus, the children with the lowest BF% had the lowest number, and the difference in BF% between this child and the second was the smallest possible. The principle of the smallest possible difference was maintained for all children, producing a sequence with gradually increasing BF%. The children were then divided into tertiles with increasing BF% (low, medium, and high, *n* = 14, 13, and 13, respectively). This ranking and grouping procedure was also carried out for BF% calculated via the 3C model. The ranges for the tertiles for BF% were: low, 13.5%–17.5% (BIA) and 15.8%–22.8% (3C model); medium, 17.6%–20.2% (BIA) and 22.9%–26.6% (3C model); and high, 20.3%–33.9% (BIA) and 26.8%–46.3% (3C model). The classification capacity for BF% estimated from BIA was then evaluated as the number of children placed in the same (0), in the next highest (+1) or lowest (−1), and in the second next highest (+2) or lowest (−2) group when compared to the groups obtained from BF% via the 3C model. Statistical significance (two-sided) was set at 5% and all analyses were conducted using SPSS version 22 (IBM, Armonk, NY, USA).

## 3. Results

Age, weight, height, and body composition variables are presented in Table 1. The 40 participating children comprised of 22 boys and 18 girls, with the sample having a wide variation in BF%, FM, and FFM.

**Figure 1:**
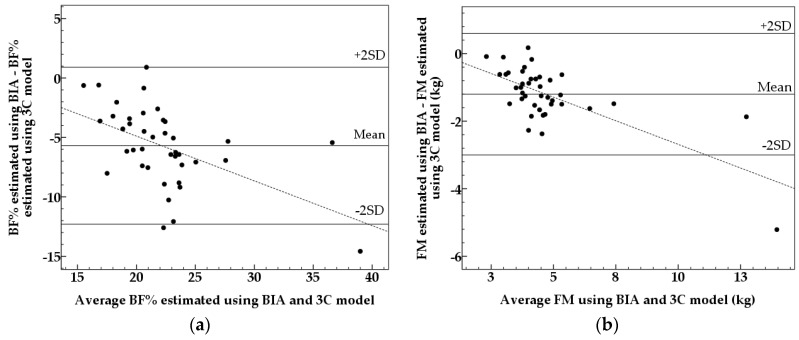

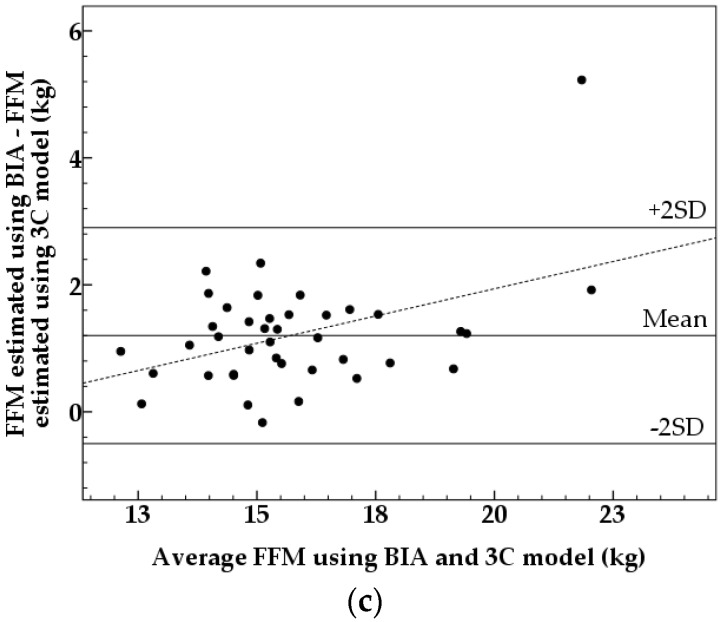
Bland and Altman plots comparing (**a**) body fat percentage (BF%) using BIA and the three component model (3C model) (mean difference: −5.7%, limits of agreement (2SD): 6.6%); (**b**) fat mass (FM) (kg) using BIA and the 3C model and (mean difference: −1.2 kg, limits of agreement (2SD): 1.8 kg) and (**c**) fat free mass (FFM) (kg) using BIA and the 3C model (mean difference: 1.2 kg, limits of agreement (2SD): 1.7 kg) in 40 healthy 5.5 years old children. The dotted line is the regression line. Regression lines: (**a**) *y* = 2.638 − 0.376*x* (*r* = −0.513, *p* = 0.001); (**b**) *y* = 0.109 − 0.280*x* (*r* = −0.705, *p* < 0.001), and (**c**) *y* = −1.495 + 0.172*x* (*r* = 0.432, *p* = 0.005).

Mean BF% and FM estimated using BIA were statistically lower (*p* < 0.001) from values calculated using the 3C model while FFM was significantly higher (*p* < 0.001). Figure 1 shows the Bland and Altman plot for BF% (Figure 1a), FM (Figure 1b,) and FFM (Figure 1c) assessed using BIA to corresponding measures determined via the 3C model. The limits of agreement were wide and significant associations were found between the average and difference for BF% (*r* = −0.513, *p* = 0.001), FM (*r* = −0.705, *p* < 0.001), and FFM (*r* = 0.432, *p* = 0.005). Significant trends were found in all three Bland and Altman plots, indicating that BF% and FM are underestimated in the children with the highest BF% and FM and FFM is overestimated in the children with the highest FFM. In comparison to the 3C model BIA underestimated BF% in 39 out of 40 children. Furthermore, as FFM is the difference of body weight minus FM, FFM was overestimated in almost all of the children using BIA.

Using Pearson correlation analyses we found that BF%, FM, and FFM were all significantly associated for BIA *versus* the 3C model (*r* = 0.809, 0.960, and 0.937, *p* < 0.001). The regression equations for BIA *versus* the 3C model were: *y* = 2.974 + 1.142*x* (*R*^2^ = 0.645, SEE = 3.29) for BF%, *y* = 0.122 + 1.265*x* (*R*^2^ = 0.919, SEE = 0.73) for FM, and *y* = 2.179 + 0.793*x* (*R*^2^ = 0.874, SEE = 0.73). When dividing the participants into tertiles (low, moderate, and high) for BF% calculated using BIA and the 3C model, 60% of the children (*n* = 24) were classified correctly. Thirty percent (*n* = 12) were classified plus or minus one group with the remaining 10% (*n* = 4) being classified plus or minus two groups.

## 4. Discussion

There are numerous different brands and types of BIA machines which makes comparisons between studies difficult. In comparison with the 3C model we found that BF% was underestimated with the Tanita SC-240 body composition analyzer. The mean difference found between BF% calculated using BIA and the 3C model was large and statistically different (−5.7 BF%). One other study in children and adolescents has evaluated the validity of the Tanita SC-240 body composition analyzer to DXA and found a mean difference of −1 BF% [6]. It is important to note that this study had a very wide age range, 5 to 18 years (mean = 10.9 ± 3.7 years) and consequently also large variations in weight (SD = 34.6 kg) and height (SD = 22.1 cm) which may have influenced their results. The children in our study were just over five years of age on average and five years is the first age for which the Tanita SC-240 is recommended for use by the manufacturer. Therefore, it is possible that the Tanita SC-240 may be better at estimating body composition more accurately at older ages.

The majority of studies to date have compared body composition variables from BIA to those obtained from DXA. Three studies in children with a mean age of 9.3, 8.9, and 8.5 years have evaluated foot-to-foot BIA in comparison with DXA and found mean differences of −4.1 [7], −0.9 (boys) and −3.3 (girls) [8], and −6.8 [18], BF% respectively. Tompuri *et al.* [19] assessed a segmental multi-frequency BIA in comparison to DXA in 7.7 years old children and found a mean difference of −4.2 and −0.3 BF% for girls and boys, respectively. To our knowledge only one other study has compared BF% using BIA and the 3C model in children. They found similar results to us with BF% being underestimated by −5.5 for girls and −4.0 BF% for boys on average [20]. Bray *et al.* [21] assessed BF% using a 4C model to BF% acquired via a varying frequency BIA and found varying results depending on the prediction equations used. However, the majority of the prediction equations underestimated BF% [21]. Within these comparisons the Tanita SC-240 body composition analyzer appears to have a comparable ability as other BIA devices to estimate BF% in young children.

As expected, the wide limits of agreement in the Bland and Altman plot show that the Tanita SC-240 is not able to predict BF%, FM, or FFM on an individual level. Wide limits of agreement for BF% and FM assessed using BIA have also been observed in other studies using DXA [6,7,8,18,19], the 3C model [20,22], and the 4C model [21] as reference methods. We also found a significant association between the mean and difference of BF% using BIA and the 3C model (*r* = −0.513, *p* = 0.001) demonstrating that the Tanita SC-240 tends to underestimate children with a high BF%, which has previously been found in other studies [7,18,19,20,21].

In regards to classification capacity, the Tanita SC-240 body composition analyzer was able to correctly classify 60% of the children in comparison to the 3C model for BF%. To our knowledge, only one other study has investigated the classification capacity of BIA in young children. Luque *et al.* [23] investigated the ability of a hand-to-foot BIA to DXA in their ability to correctly classify seven years old children into quartiles based on their fat mass index. They found that 71.3% of children were correctly classified when using BIA outputs [23]. In our study, when we compared fat mass index assessed using BIA to the 3C model we were able to correctly classify 50% of the subjects using tertiles. The better ranking capacity found by Luque *et al.* [23] is probably due to the type of BIA used (hand-to-foot *vs.* foot-to-foot). The foot-to-foot models, for example the Tanita SC-240 only measures resistance in the lower part of the body whereas the hand-to-foot models measures resistance in both the lower and upper body [24].

In this study we compared body composition variables assessed with BIA to the 3C model. The advantage of using the 3C model over 2C models such as DXA or ADP is that TBW is measured and the assumptions for hydration of FFM are eliminated [25]. Using the 3C model FFM is then divided into water and dry FFM, which is mainly made up of protein and mineral [14]. Another option would have been to use the 4C model as a reference since it is even more superior as it divides the FFM into water, protein, and mineral [14]. However, this would add another dimension (measurement of bone mineral with DXA) that did not fit within the MINISTOP trial design, as it already had an extensive protocol with several physiological measurements. It is important to highlight that it has been found that the contribution of mineral to the model is relatively minor in comparison to TBW and body volume [14]. Furthermore, in this context it is relevant to note that Wells *et al.* [25] compared the 3C to the 4C model in 8 to 12 years old children and found no significant difference in BF%.

Simple anthropometric measurements such as BMI have been found to be inadequate when measuring obesity in young children [3,4]. Forsum *et al.* [4] found that BMI was only able to explain about 15% of the variation in BF%. In this study we were able to explain just over 65% of the variation in BF% using BIA. Although this indicates a relatively high ranking capacity, in absolute values the Tanita SC-240 underestimated BF% with a systematic bias with increased underestimation in children with high BF% levels. Further work is needed in order to develop field methods for body composition with acceptable accuracy that can be scaled up for epidemiological studies.

Strengths of this study include the use of the 3C model as a reference method as well as the use of a homogenous population in terms of age and ethnicity. This study is limited by the unavailability of the raw BIA variables as well as its small sample size. Therefore, further studies in larger populations are warranted.

## 5. Conclusions

In conclusion, the Tanita underestimated BF% on average in comparison to the 3C model, and demonstrated poor ability to estimate BF% in individuals. Further work is needed in order to find accurate and easy-to-use methods to assess body composition in pre-school children.

## Figures and Tables

**Table 1:** Age, weight, and height as well as body composition variables by means of bioelectrical impedance analysis (BIA) and three component (3C) model for the participating children (*n* = 40).

Variable	BIA		3C Model	
	Mean ± SD	Range	Mean ± SD	Range
Age (years)	5.5 ± 0.2	5.2–5.7	-	-
Weight (kg)	20.5 ± 4.2	14.9–35.8	-	-
Weight for age *z*-score ^1^	−0.05 ± 1.55	−2.22–5.41	-	-
Height (cm)	114.0 ± 4.0	105.0–125.5	-	-
Height for age *z*-score ^1^	0.00 ± 0.90	−1.92–2.26	-	-
BMI (kg/m^2^) ^2^	15.6 ± 2.3	13.3–25.6	-	-
Body fat percentage	19.4 ± 3.9 *	13.5–33.9	25.1 ± 5.5	15.8–46.3
Fat mass (kg)	4.1 ± 1.9 *	2.3–11.8	5.3 ± 2.5	2.4–16.6
Fat free mass (kg)	16.4 ± 2.4 *	12.6–24.5	15.2 ± 2.0	11.7–21.1
FMI (kg/m^2^)	3.1 ± 1.2 *	1.8–8.1	4.0 ± 1.7	2.1–11.8
FFMI (kg/m^2^)	12.5 ± 1.1 *	10.8–17.5	11.6 ± 0.8	10.0–13.7

BIA, bioelectrical impedance; 3C Model, 3 component model; FMI; fat mass index; FFMI, fat free mass index; ^1^ Calculated using Swedish reference data [13]; ^2^ One child was classified as overweight and two as obese [12]; * Significantly different from the corresponding value obtained using the 3C model (*p* < 0.001).

## Abbreviations

The following abbreviations are used in this manuscript:

BMI	Body Mass Index
BF%	Body Fat Percentage
FM	Fat Mass
FFM	Fat Free Mass
TBW	Total Body Water
ADP	Air Displacement Plethysmography
BIA	Bioelectrical Impedance
DXA	Dual Energy X-ray Absorptiometry
2C Model	Two Component Model
3C Model	Three Component Model
4C Model	Four Component Model
SD	Standard Deviation
